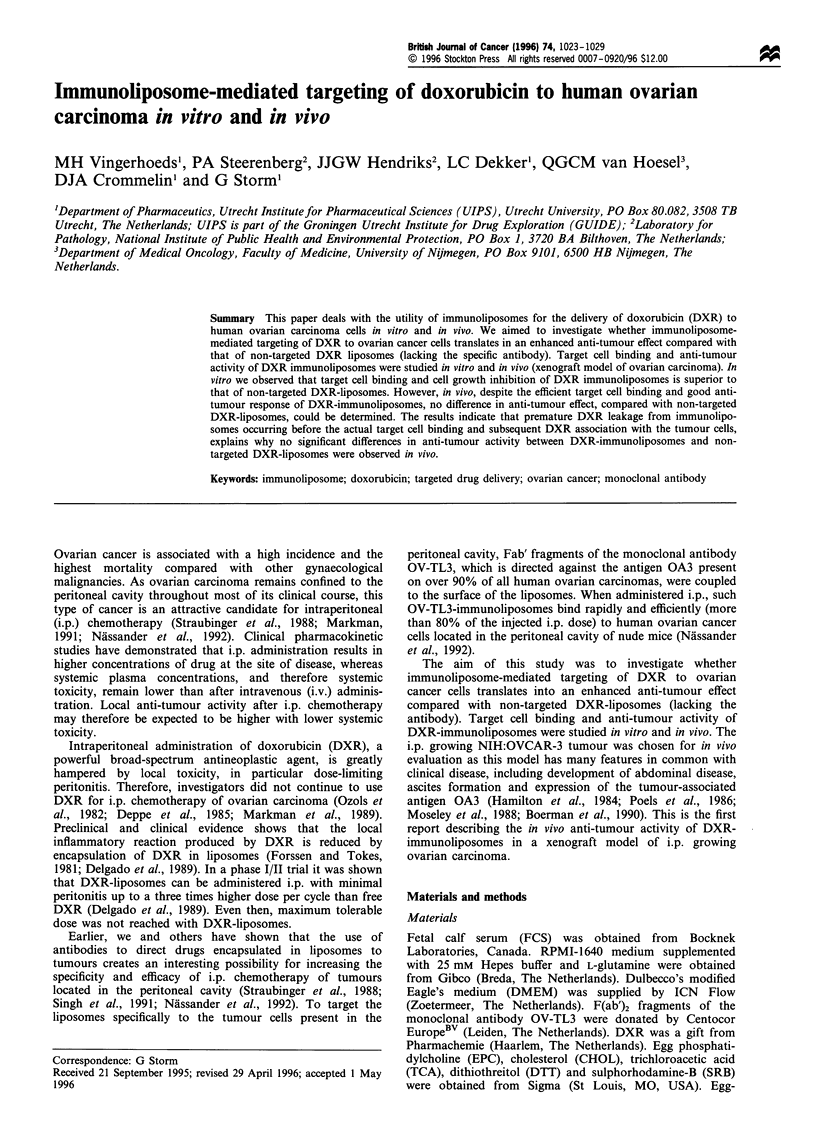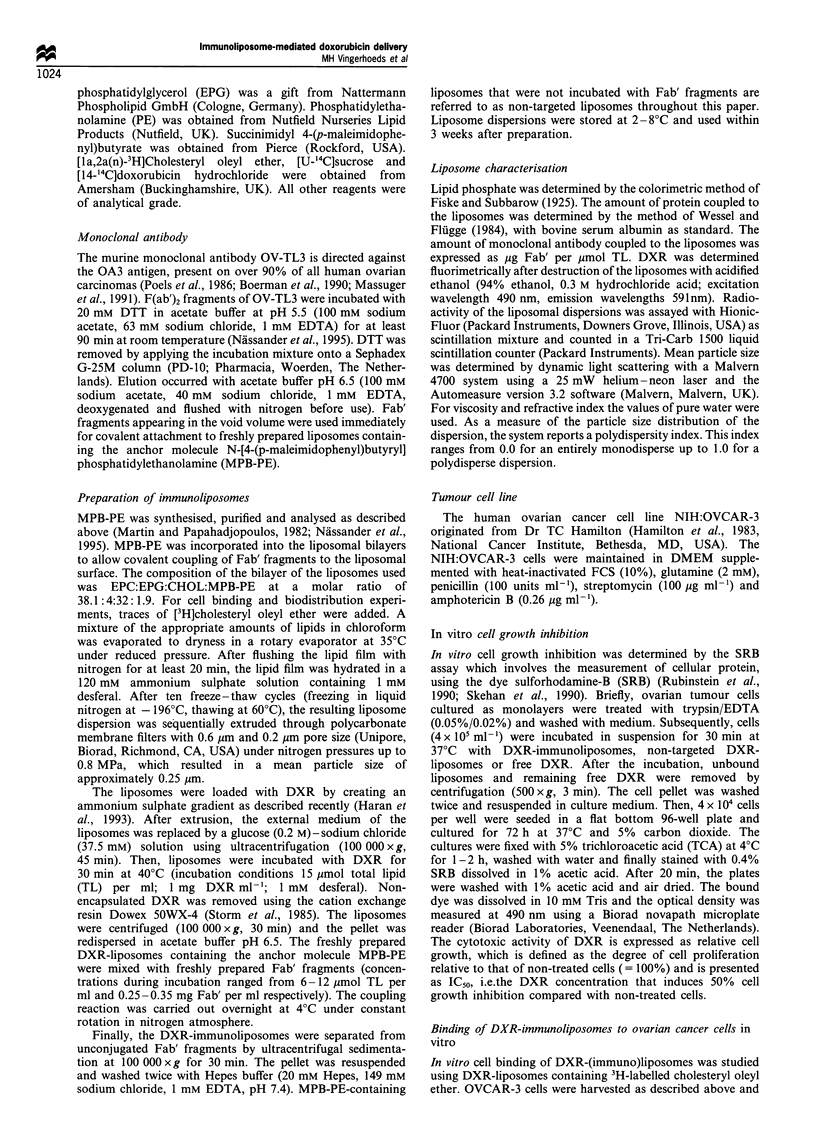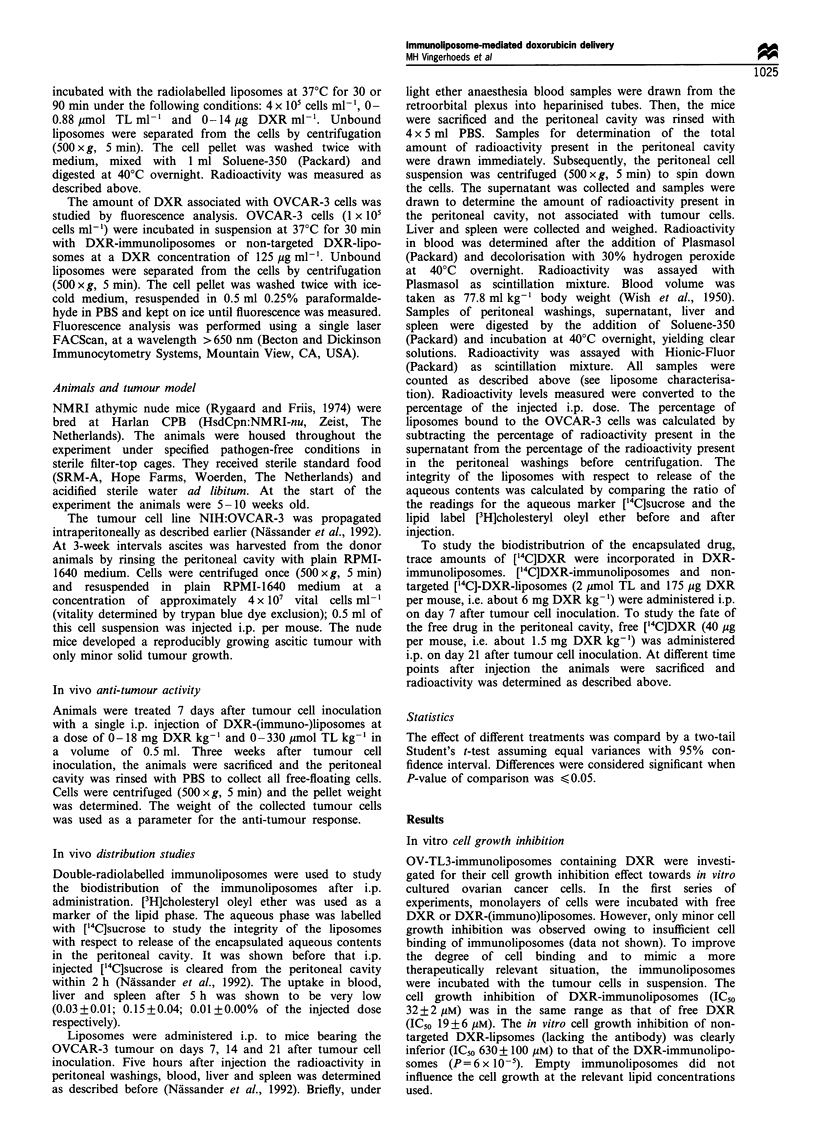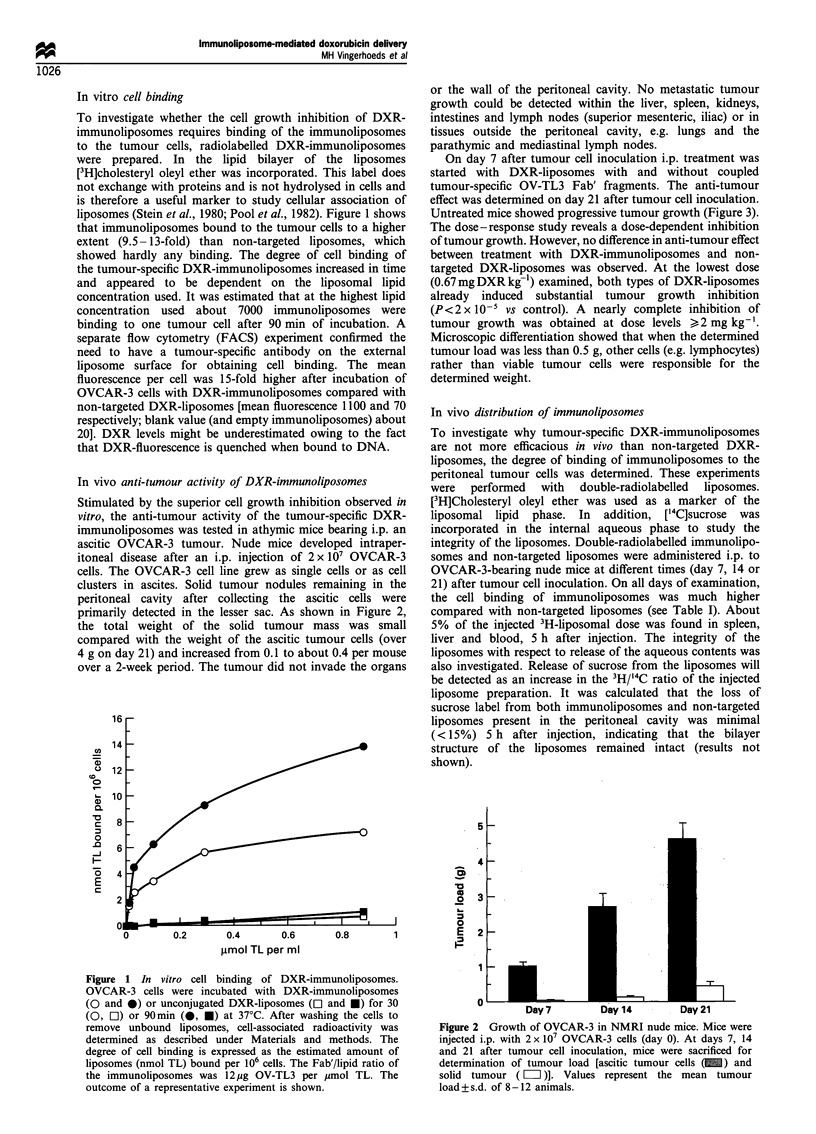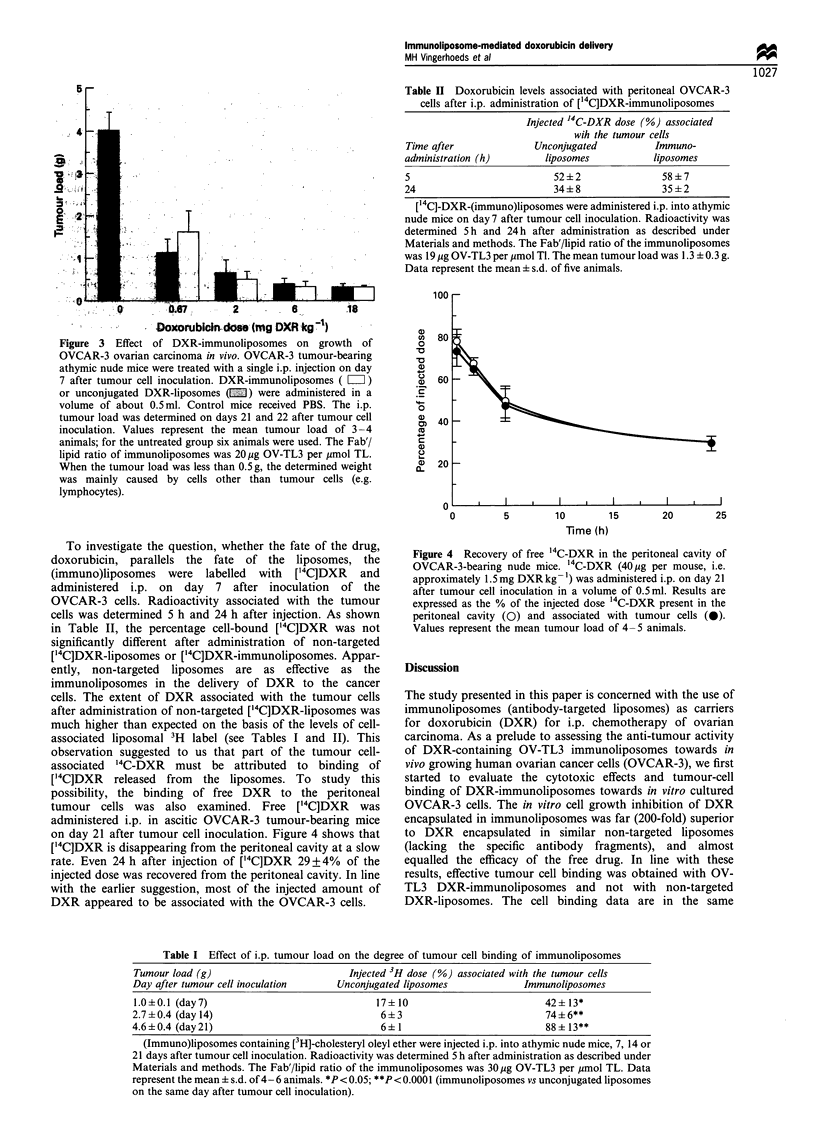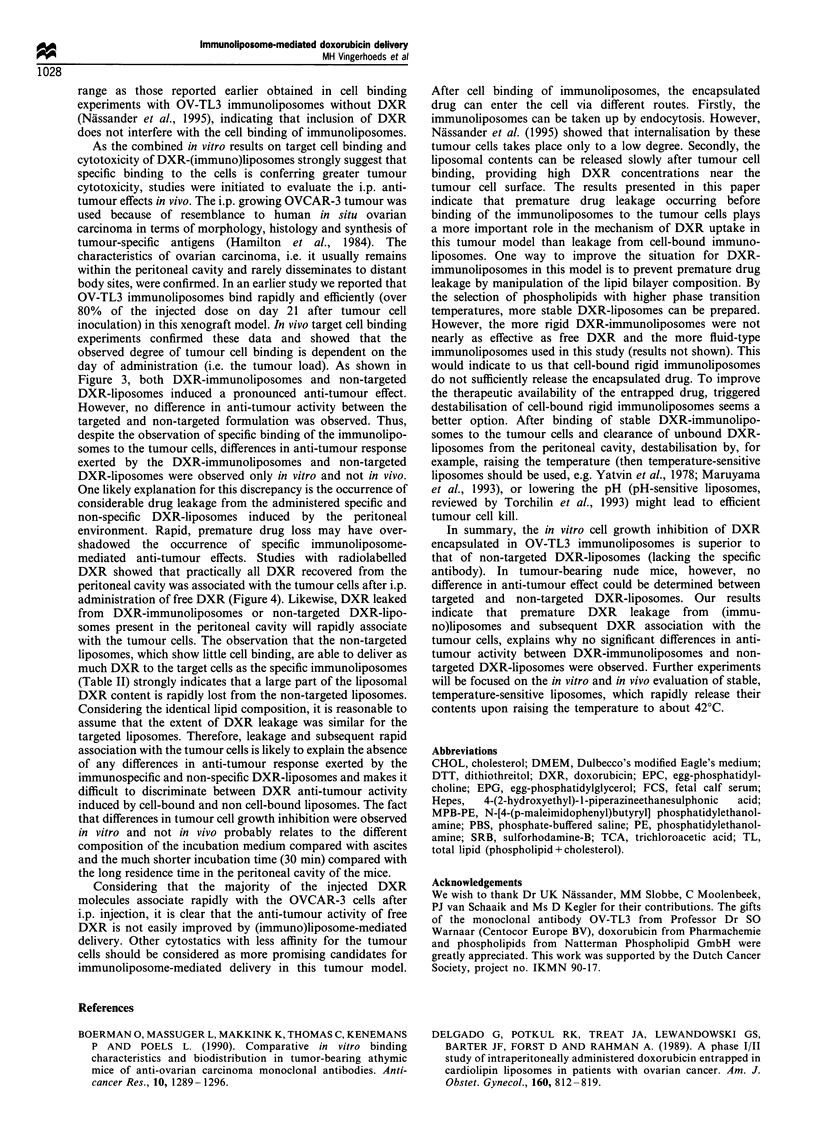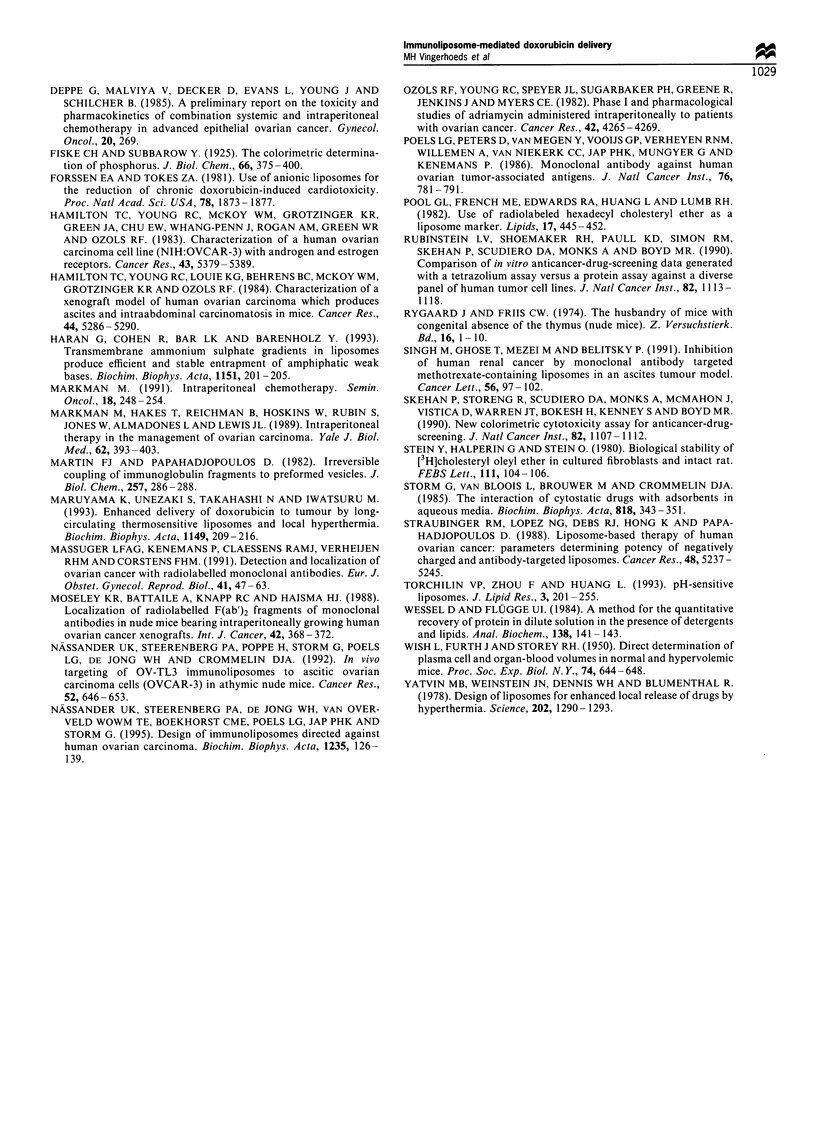# Immunoliposome-mediated targeting of doxorubicin to human ovarian carcinoma in vitro and in vivo.

**DOI:** 10.1038/bjc.1996.484

**Published:** 1996-10

**Authors:** M. H. Vingerhoeds, P. A. Steerenberg, J. J. Hendriks, L. C. Dekker, Q. G. Van Hoesel, D. J. Crommelin, G. Storm

**Affiliations:** Department of Pharmaceutics, Utrecht Institute for Pharmaceutical Sciences (UIPS), Utrecht University, The Netherlands.

## Abstract

This paper deals with the utility of immunoliposomes for the delivery of doxorubicin (DXR) to human ovarian carcinoma cells in vitro and in vivo. We aimed to investigate whether immunoliposome-mediated targeting of DXR to ovarian cancer cells translates in an enhanced anti-tumour effect compared with that of non-targeted DXR liposomes (lacking the specific antibody). Target cell binding and anti-tumour activity of DXR immunoliposomes were studied in vitro and in vivo (xenograft model of ovarian carcinoma). In vitro we observed that target cell binding and cell growth inhibition of DXR immunoliposomes is superior to that of non-targeted DXR-liposomes. However, in vivo, despite the efficient target cell binding and good anti-tumour response of DXR-immunoliposomes, no difference in anti-tumour effect, compared with non-targeted DXR-liposomes, could be determined. The results indicate that premature DXR leakage from immunoliposomes occurring before the actual target cell binding and subsequent DXR association with the tumour cells, explains why no significant differences in anti-tumour activity between DXR-immunoliposomes and non-targeted DXR-liposomes were observed in vivo.


British Journal of Cancer (1996) 74, 1023-1029
© 1996 Stockton Press All rights reserved 0007-0920/96 $12.00

### Immunoliposome-mediated targeting of doxorubicin to human ovarian carcinoma *in vitro* and *in vivo*

MH Vingerhoeds[1], PA Steerenberg[2], JJGW Hendriks[2], LC Dekker[1], QGCM van Hoesel[3], DJA Crommelin[1] and G Storm[1]

[1]*Department of Pharmaceutics, Utrecht Institute for Pharmaceutical Sciences (UIPS), Utrecht University, PO Box 80.082, 3508 TB Utrecht, The Netherlands; UIPS is part of the Groningen Utrecht Institute for Drug Exploration (GUIDE);* [2]*Laboratory for Pathology, National Institute of Public Health and Environmental Protection, PO Box 1, 3720 BA Bilthoven, The Netherlands;* [3]*Department of Medical Oncology, Faculty of Medicine, University of Nijmegen, PO Box 9101, 6500 HB Nijmegen, The Netherlands.*

**Summary** This paper deals with the utility of immunoliposomes for the delivery of doxorubicin (DXR) to human ovarian carcinoma cells *in vitro* and *in vivo*. We aimed to investigate whether immunoliposome-mediated targeting of DXR to ovarian cancer cells translates in an enhanced anti-tumour effect compared with that of non-targeted DXR liposomes (lacking the specific antibody). Target cell binding and anti-tumour activity of DXR immunoliposomes were studied *in vitro* and *in vivo* (xenograft model of ovarian carcinoma). *In vitro* we observed that target cell binding and cell growth inhibition of DXR immunoliposomes is superior to that of non-targeted DXR-liposomes. However, *in vivo*, despite the efficient target cell binding and good anti-tumour response of DXR-immunoliposomes, no difference in anti-tumour effect, compared with non-targeted DXR-liposomes, could be determined. The results indicate that premature DXR leakage from immunoliposomes occurring before the actual target cell binding and subsequent DXR association with the tumour cells, explains why no significant differences in anti-tumour activity between DXR-immunoliposomes and non-targeted DXR-liposomes were observed *in vivo*.

**Keywords:** immunoliposome; doxorubicin; targeted drug delivery; ovarian cancer; monoclonal antibody

Ovarian cancer is associated with a high incidence and the highest mortality compared with other gynaecological malignancies. As ovarian carcinoma remains confined to the peritoneal cavity throughout most of its clinical course, this type of cancer is an attractive candidate for intraperitoneal (i.p.) chemotherapy (Straubinger *et al.*, 1988; Markman, 1991; Nässander *et al.*, 1992). Clinical pharmacokinetic studies have demonstrated that i.p. administration results in higher concentrations of drug at the site of disease, whereas systemic plasma concentrations, and therefore systemic toxicity, remain lower than after intravenous (i.v.) administration. Local anti-tumour activity after i.p. chemotherapy may therefore be expected to be higher with lower systemic toxicity.

Intraperitoneal administration of doxorubicin (DXR), a powerful broad-spectrum antineoplastic agent, is greatly hampered by local toxicity, in particular dose-limiting peritonitis. Therefore, investigators did not continue to use DXR for i.p. chemotherapy of ovarian carcinoma (Ozols *et al.*, 1982; Deppe *et al.*, 1985; Markman *et al.*, 1989). Preclinical and clinical evidence shows that the local inflammatory reaction produced by DXR is reduced by encapsulation of DXR in liposomes (Forssen and Tokes, 1981; Delgado *et al.*, 1989). In a phase I/II trial it was shown that DXR-liposomes can be administered i.p. with minimal peritonitis up to a three times higher dose per cycle than free DXR (Delgado *et al.*, 1989). Even then, maximum tolerable dose was not reached with DXR-liposomes.

Earlier, we and others have shown that the use of antibodies to direct drugs encapsulated in liposomes to tumours creates an interesting possibility for increasing the specificity and efficacy of i.p. chemotherapy of tumours located in the peritoneal cavity (Straubinger *et al.*, 1988; Singh *et al.*, 1991; Nässander *et al.*, 1992). To target the liposomes specifically to the tumour cells present in the peritoneal cavity, Fab′ fragments of the monoclonal antibody OV-TL3, which is directed against the antigen OA3 present on over 90% of all human ovarian carcinomas, were coupled to the surface of the liposomes. When administered i.p., such OV-TL3-immunoliposomes bind rapidly and efficiently (more than 80% of the injected i.p. dose) to human ovarian cancer cells located in the peritoneal cavity of nude mice (Nässander *et al.*, 1992).

The aim of this study was to investigate whether immunoliposome-mediated targeting of DXR to ovarian cancer cells translates into an enhanced anti-tumour effect compared with non-targeted DXR-liposomes (lacking the antibody). Target cell binding and anti-tumour activity of DXR-immunoliposomes were studied *in vitro* and *in vivo*. The i.p. growing NIH:OVCAR-3 tumour was chosen for *in vivo* evaluation as this model has many features in common with clinical disease, including development of abdominal disease, ascites formation and expression of the tumour-associated antigen OA3 (Hamilton *et al.*, 1984; Poels *et al.*, 1986; Moseley *et al.*, 1988; Boerman *et al.*, 1990). This is the first report describing the *in vivo* anti-tumour activity of DXR-immunoliposomes in a xenograft model of i.p. growing ovarian carcinoma.

### Materials and methods

### *Materials*

Fetal calf serum (FCS) was obtained from Bocknek Laboratories, Canada. RPMI-1640 medium supplemented with 25 mM Hepes buffer and L-glutamine were obtained from Gibco (Breda, The Netherlands). Dulbecco's modified Eagle's medium (DMEM) was supplied by ICN Flow (Zoetermeer, The Netherlands). $F(ab')_2$ fragments of the monoclonal antibody OV-TL3 were donated by Centocor Europe[BV] (Leiden, The Netherlands). DXR was a gift from Pharmachemie (Haarlem, The Netherlands). Egg phosphatidylcholine (EPC), cholesterol (CHOL), trichloroacetic acid (TCA), dithiothreitol (DTT) and sulphorhodamine-B (SRB) were obtained from Sigma (St Louis, MO, USA). Egg-

Correspondence: G Storm
Received 21 September 1995; revised 29 April 1996; accepted 1 May 1996

phosphatidylglycerol (EPG) was a gift from Nattermann Phospholipid GmbH (Cologne, Germany). Phosphatidylethanolamine (PE) was obtained from Nutfield Nurseries Lipid Products (Nutfield, UK). Succinimidyl 4-(*p*-maleimidophenyl)butyrate was obtained from Pierce (Rockford, USA). [1a,2a(n)-$^{3}$H]Cholesteryl oleyl ether, [U-$^{14}$C]sucrose and [14-$^{14}$C]doxorubicin hydrochloride were obtained from Amersham (Buckinghamshire, UK). All other reagents were of analytical grade.

### *Monoclonal antibody*

The murine monoclonal antibody OV-TL3 is directed against the OA3 antigen, present on over 90% of all human ovarian carcinomas (Poels *et al*., 1986; Boerman *et al*., 1990; Massuger *et al*., 1991). F(ab')$_2$ fragments of OV-TL3 were incubated with 20 mM DTT in acetate buffer at pH 5.5 (100 mM sodium acetate, 63 mM sodium chloride, 1 mM EDTA) for at least 90 min at room temperature (Nässander *et al*., 1995). DTT was removed by applying the incubation mixture onto a Sephadex G-25M column (PD-10; Pharmacia, Woerden, The Netherlands). Elution occurred with acetate buffer pH 6.5 (100 mM sodium acetate, 40 mM sodium chloride, 1 mM EDTA, deoxygenated and flushed with nitrogen before use). Fab' fragments appearing in the void volume were used immediately for covalent attachment to freshly prepared liposomes containing the anchor molecule N-[4-(p-maleimidophenyl)butyryl] phosphatidylethanolamine (MPB-PE).

### *Preparation of immunoliposomes*

MPB-PE was synthesised, purified and analysed as described above (Martin and Papahadjopoulos, 1982; Nässander *et al*., 1995). MPB-PE was incorporated into the liposomal bilayers to allow covalent coupling of Fab' fragments to the liposomal surface. The composition of the bilayer of the liposomes used was EPC:EPG:CHOL:MPB-PE at a molar ratio of 38.1:4:32:1.9. For cell binding and biodistribution experiments, traces of [$^{3}$H]cholesteryl oleyl ether were added. A mixture of the appropriate amounts of lipids in chloroform was evaporated to dryness in a rotary evaporator at 35°C under reduced pressure. After flushing the lipid film with nitrogen for at least 20 min, the lipid film was hydrated in a 120 mM ammonium sulphate solution containing 1 mM desferal. After ten freeze–thaw cycles (freezing in liquid nitrogen at −196°C, thawing at 60°C), the resulting liposome dispersion was sequentially extruded through polycarbonate membrane filters with 0.6 $\mu$m and 0.2 $\mu$m pore size (Unipore, Biorad, Richmond, CA, USA) under nitrogen pressures up to 0.8 MPa, which resulted in a mean particle size of approximately 0.25 $\mu$m.

The liposomes were loaded with DXR by creating an ammonium sulphate gradient as described recently (Haran *et al*., 1993). After extrusion, the external medium of the liposomes was replaced by a glucose (0.2 M)–sodium chloride (37.5 mM) solution using ultracentrifugation (100 000 × *g*, 45 min). Then, liposomes were incubated with DXR for 30 min at 40°C (incubation conditions 15 $\mu$mol total lipid (TL) per ml; 1 mg DXR ml$^{-1}$; 1 mM desferal). Non-encapsulated DXR was removed using the cation exchange resin Dowex 50WX-4 (Storm *et al*., 1985). The liposomes were centrifuged (100 000 × *g*, 30 min) and the pellet was redispersed in acetate buffer pH 6.5. The freshly prepared DXR-liposomes containing the anchor molecule MPB-PE were mixed with freshly prepared Fab' fragments (concentrations during incubation ranged from 6–12 $\mu$mol TL per ml and 0.25–0.35 mg Fab' per ml respectively). The coupling reaction was carried out overnight at 4°C under constant rotation in nitrogen atmosphere.

Finally, the DXR-immunoliposomes were separated from unconjugated Fab' fragments by ultracentrifugal sedimentation at 100 000 × *g* for 30 min. The pellet was resuspended and washed twice with Hepes buffer (20 mM Hepes, 149 mM sodium chloride, 1 mM EDTA, pH 7.4). MPB-PE-containing liposomes that were not incubated with Fab' fragments are referred to as non-targeted liposomes throughout this paper. Liposome dispersions were stored at 2–8°C and used within 3 weeks after preparation.

### *Liposome characterisation*

Lipid phosphate was determined by the colorimetric method of Fiske and Subbarow (1925). The amount of protein coupled to the liposomes was determined by the method of Wessel and Flügge (1984), with bovine serum albumin as standard. The amount of monoclonal antibody coupled to the liposomes was expressed as $\mu$g Fab' per $\mu$mol TL. DXR was determined fluorimetrically after destruction of the liposomes with acidified ethanol (94% ethanol, 0.3 M hydrochloride acid; excitation wavelength 490 nm, emission wavelengths 591nm). Radioactivity of the liposomal dispersions was assayed with Hionic-Fluor (Packard Instruments, Downers Grove, Illinois, USA) as scintillation mixture and counted in a Tri-Carb 1500 liquid scintillation counter (Packard Instruments). Mean particle size was determined by dynamic light scattering with a Malvern 4700 system using a 25 mW helium–neon laser and the Automeasure version 3.2 software (Malvern, Malvern, UK). For viscosity and refractive index the values of pure water were used. As a measure of the particle size distribution of the dispersion, the system reports a polydispersity index. This index ranges from 0.0 for an entirely monodisperse up to 1.0 for a polydisperse dispersion.

### *Tumour cell line*

The human ovarian cancer cell line NIH:OVCAR-3 originated from Dr TC Hamilton (Hamilton *et al*., 1983, National Cancer Institute, Bethesda, MD, USA). The NIH:OVCAR-3 cells were maintained in DMEM supplemented with heat-inactivated FCS (10%), glutamine (2 mM), penicillin (100 units ml$^{-1}$), streptomycin (100 $\mu$g ml$^{-1}$) and amphotericin B (0.26 $\mu$g ml$^{-1}$).

### In vitro *cell growth inhibition*

*In vitro* cell growth inhibition was determined by the SRB assay which involves the measurement of cellular protein, using the dye sulforhodamine-B (SRB) (Rubinstein *et al*., 1990; Skehan *et al*., 1990). Briefly, ovarian tumour cells cultured as monolayers were treated with trypsin/EDTA (0.05%/0.02%) and washed with medium. Subsequently, cells ($4 \times 10^{5}$ ml$^{-1}$) were incubated in suspension for 30 min at 37°C with DXR-immunoliposomes, non-targeted DXR-liposomes or free DXR. After the incubation, unbound liposomes and remaining free DXR were removed by centrifugation (500 × *g*, 3 min). The cell pellet was washed twice and resuspended in culture medium. Then, $4 \times 10^{4}$ cells per well were seeded in a flat bottom 96-well plate and cultured for 72 h at 37°C and 5% carbon dioxide. The cultures were fixed with 5% trichloroacetic acid (TCA) at 4°C for 1–2 h, washed with water and finally stained with 0.4% SRB dissolved in 1% acetic acid. After 20 min, the plates were washed with 1% acetic acid and air dried. The bound dye was dissolved in 10 mM Tris and the optical density was measured at 490 nm using a Biorad novapath microplate reader (Biorad Laboratories, Veenendaal, The Netherlands). The cytotoxic activity of DXR is expressed as relative cell growth, which is defined as the degree of cell proliferation relative to that of non-treated cells (= 100%) and is presented as $IC_{50}$, i.e.the DXR concentration that induces 50% cell growth inhibition compared with non-treated cells.

### *Binding of DXR-immunoliposomes to ovarian cancer cells* in vitro

*In vitro* cell binding of DXR-(immuno)liposomes was studied using DXR-liposomes containing $^{3}$H-labelled cholesteryl oleyl ether. OVCAR-3 cells were harvested as described above and incubated with the radiolabelled liposomes at 37°C for 30 or 90 min under the following conditions: $4\times10^5$ cells $ml^{-1}$, 0–0.88 $\mu$mol TL $ml^{-1}$ and 0–14 $\mu$g DXR $ml^{-1}$. Unbound liposomes were separated from the cells by centrifugation (500 × ***g***, 5 min). The cell pellet was washed twice with medium, mixed with 1 ml Soluene-350 (Packard) and digested at 40°C overnight. Radioactivity was measured as described above.

The amount of DXR associated with OVCAR-3 cells was studied by fluorescence analysis. OVCAR-3 cells ($1\times10^5$ cells $ml^{-1}$) were incubated in suspension at 37°C for 30 min with DXR-immunoliposomes or non-targeted DXR-liposomes at a DXR concentration of 125 $\mu$g $ml^{-1}$. Unbound liposomes were separated from the cells by centrifugation (500 × ***g***, 5 min). The cell pellet was washed twice with ice-cold medium, resuspended in 0.5 ml 0.25% paraformaldehyde in PBS and kept on ice until fluorescence was measured. Fluorescence analysis was performed using a single laser FACScan, at a wavelength >650 nm (Becton and Dickinson Immunocytometry Systems, Mountain View, CA, USA).

### *Animals and tumour model*

NMRI athymic nude mice (Rygaard and Friis, 1974) were bred at Harlan CPB (HsdCpn:NMRI-*nu*, Zeist, The Netherlands). The animals were housed throughout the experiment under specified pathogen-free conditions in sterile filter-top cages. They received sterile standard food (SRM-A, Hope Farms, Woerden, The Netherlands) and acidified sterile water *ad libitum*. At the start of the experiment the animals were 5–10 weeks old.

The tumour cell line NIH:OVCAR-3 was propagated intraperitoneally as described earlier (Nässander *et al.*, 1992). At 3-week intervals ascites was harvested from the donor animals by rinsing the peritoneal cavity with plain RPMI-1640 medium. Cells were centrifuged once (500 × ***g***, 5 min) and resuspended in plain RPMI-1640 medium at a concentration of approximately $4\times10^7$ vital cells $ml^{-1}$ (vitality determined by trypan blue dye exclusion); 0.5 ml of this cell suspension was injected i.p. per mouse. The nude mice developed a reproducibly growing ascitic tumour with only minor solid tumour growth.

### In vivo *anti-tumour activity*

Animals were treated 7 days after tumour cell inoculation with a single i.p. injection of DXR-(immuno-)liposomes at a dose of 0–18 mg DXR $kg^{-1}$ and 0–330 $\mu$mol TL $kg^{-1}$ in a volume of 0.5 ml. Three weeks after tumour cell inoculation, the animals were sacrificed and the peritoneal cavity was rinsed with PBS to collect all free-floating cells. Cells were centrifuged (500 × ***g***, 5 min) and the pellet weight was determined. The weight of the collected tumour cells was used as a parameter for the anti-tumour response.

### In vivo *distribution studies*

Double-radiolabelled immunoliposomes were used to study the biodistribution of the immunoliposomes after i.p. administration. [$^3$H]cholesteryl oleyl ether was used as a marker of the lipid phase. The aqueous phase was labelled with [$^{14}$C]sucrose to study the integrity of the liposomes with respect to release of the encapsulated aqueous contents in the peritoneal cavity. It was shown before that i.p. injected [$^{14}$C]sucrose is cleared from the peritoneal cavity within 2 h (Nässander *et al.*, 1992). The uptake in blood, liver and spleen after 5 h was shown to be very low ($0.03\pm0.01$; $0.15\pm0.04$; $0.01\pm0.00$% of the injected dose respectively).

Liposomes were administered i.p. to mice bearing the OVCAR-3 tumour on days 7, 14 and 21 after tumour cell inoculation. Five hours after injection the radioactivity in peritoneal washings, blood, liver and spleen was determined as described before (Nässander *et al.*, 1992). Briefly, under light ether anaesthesia blood samples were drawn from the retroorbital plexus into heparinised tubes. Then, the mice were sacrificed and the peritoneal cavity was rinsed with 4 × 5 ml PBS. Samples for determination of the total amount of radioactivity present in the peritoneal cavity were drawn immediately. Subsequently, the peritoneal cell suspension was centrifuged (500 × ***g***, 5 min) to spin down the cells. The supernatant was collected and samples were drawn to determine the amount of radioactivity present in the peritoneal cavity, not associated with tumour cells. Liver and spleen were collected and weighed. Radioactivity in blood was determined after the addition of Plasmasol (Packard) and decolorisation with 30% hydrogen peroxide at 40°C overnight. Radioactivity was assayed with Plasmasol as scintillation mixture. Blood volume was taken as 77.8 ml $kg^{-1}$ body weight (Wish *et al.*, 1950). Samples of peritoneal washings, supernatant, liver and spleen were digested by the addition of Soluene-350 (Packard) and incubation at 40°C overnight, yielding clear solutions. Radioactivity was assayed with Hionic-Fluor (Packard) as scintillation mixture. All samples were counted as described above (see liposome characterisation). Radioactivity levels measured were converted to the percentage of the injected i.p. dose. The percentage of liposomes bound to the OVCAR-3 cells was calculated by subtracting the percentage of radioactivity present in the supernatant from the percentage of the radioactivity present in the peritoneal washings before centrifugation. The integrity of the liposomes with respect to release of the aqueous contents was calculated by comparing the ratio of the readings for the aqueous marker [$^{14}$C]sucrose and the lipid label [$^3$H]cholesteryl oleyl ether before and after injection.

To study the biodistributrion of the encapsulated drug, trace amounts of [$^{14}$C]DXR were incorporated in DXR-immunoliposomes. [$^{14}$C]DXR-immunoliposomes and non-targeted [$^{14}$C]-DXR-liposomes (2 $\mu$mol TL and 175 $\mu$g DXR per mouse, i.e. about 6 mg DXR $kg^{-1}$) were administered i.p. on day 7 after tumour cell inoculation. To study the fate of the free drug in the peritoneal cavity, free [$^{14}$C]DXR (40 $\mu$g per mouse, i.e. about 1.5 mg DXR $kg^{-1}$) was administered i.p. on day 21 after tumour cell inoculation. At different time points after injection the animals were sacrificed and radioactivity was determined as described above.

### *Statistics*

The effect of different treatments was compard by a two-tail Student's *t*-test assuming equal variances with 95% confidence interval. Differences were considered significant when *P*-value of comparison was $\leqslant 0.05$.

### Results

### In vitro *cell growth inhibition*

OV-TL3-immunoliposomes containing DXR were investigated for their cell growth inhibition effect towards *in vitro* cultured ovarian cancer cells. In the first series of experiments, monolayers of cells were incubated with free DXR or DXR-(immuno)liposomes. However, only minor cell growth inhibition was observed owing to insufficient cell binding of immunoliposomes (data not shown). To improve the degree of cell binding and to mimic a more therapeutically relevant situation, the immunoliposomes were incubated with the tumour cells in suspension. The cell growth inhibition of DXR-immunoliposomes ($IC_{50}$ $32\pm2$ $\mu$M) was in the same range as that of free DXR ($IC_{50}$ $19\pm6$ $\mu$M). The *in vitro* cell growth inhibition of non-targeted DXR-lipsomes (lacking the antibody) was clearly inferior ($IC_{50}$ $630\pm100$ $\mu$M) to that of the DXR-immunoliposomes ($P=6\times10^{-5}$). Empty immunoliposomes did not influence the cell growth at the relevant lipid concentrations used.

### In vitro *cell binding*

To investigate whether the cell growth inhibition of DXR-immunoliposomes requires binding of the immunoliposomes to the tumour cells, radiolabelled DXR-immunoliposomes were prepared. In the lipid bilayer of the liposomes [$^3$H]cholesteryl oleyl ether was incorporated. This label does not exchange with proteins and is not hydrolysed in cells and is therefore a useful marker to study cellular association of liposomes (Stein *et al.*, 1980; Pool *et al.*, 1982). Figure 1 shows that immunoliposomes bound to the tumour cells to a higher extent (9.5–13-fold) than non-targeted liposomes, which showed hardly any binding. The degree of cell binding of the tumour-specific DXR-immunoliposomes increased in time and appeared to be dependent on the liposomal lipid concentration used. It was estimated that at the highest lipid concentration used about 7000 immunoliposomes were binding to one tumour cell after 90 min of incubation. A separate flow cytometry (FACS) experiment confirmed the need to have a tumour-specific antibody on the external liposome surface for obtaining cell binding. The mean fluorescence per cell was 15-fold higher after incubation of OVCAR-3 cells with DXR-immunoliposomes compared with non-targeted DXR-liposomes [mean fluorescence 1100 and 70 respectively; blank value (and empty immunoliposomes) about 20]. DXR levels might be underestimated owing to the fact that DXR-fluorescence is quenched when bound to DNA.

### In vivo *anti-tumour activity of DXR-immunoliposomes*

Stimulated by the superior cell growth inhibition observed *in vitro*, the anti-tumour activity of the tumour-specific DXR-immunoliposomes was tested in athymic mice bearing i.p. an ascitic OVCAR-3 tumour. Nude mice developed intraperitoneal disease after an i.p. injection of $2\times10^7$ OVCAR-3 cells. The OVCAR-3 cell line grew as single cells or as cell clusters in ascites. Solid tumour nodules remaining in the peritoneal cavity after collecting the ascitic cells were primarily detected in the lesser sac. As shown in Figure 2, the total weight of the solid tumour mass was small compared with the weight of the ascitic tumour cells (over 4 g on day 21) and increased from 0.1 to about 0.4 per mouse over a 2-week period. The tumour did not invade the organs or the wall of the peritoneal cavity. No metastatic tumour growth could be detected within the liver, spleen, kidneys, intestines and lymph nodes (superior mesenteric, iliac) or in tissues outside the peritoneal cavity, e.g. lungs and the parathymic and mediastinal lymph nodes.

On day 7 after tumour cell inoculation i.p. treatment was started with DXR-liposomes with and without coupled tumour-specific OV-TL3 Fab′ fragments. The anti-tumour effect was determined on day 21 after tumour cell inoculation. Untreated mice showed progressive tumour growth (Figure 3). The dose–response study reveals a dose-dependent inhibition of tumour growth. However, no difference in anti-tumour effect between treatment with DXR-immunoliposomes and non-targeted DXR-liposomes was observed. At the lowest dose (0.67 mg DXR kg$^{-1}$) examined, both types of DXR-liposomes already induced substantial tumour growth inhibition ($P<2\times10^{-5}$ *vs* control). A nearly complete inhibition of tumour growth was obtained at dose levels $\geqslant 2$ mg kg$^{-1}$. Microscopic differentiation showed that when the determined tumour load was less than 0.5 g, other cells (e.g. lymphocytes) rather than viable tumour cells were responsible for the determined weight.

### In vivo *distribution of immunoliposomes*

To investigate why tumour-specific DXR-immunoliposomes are not more efficacious *in vivo* than non-targeted DXR-liposomes, the degree of binding of immunoliposomes to the peritoneal tumour cells was determined. These experiments were performed with double-radiolabelled liposomes. [$^3$H]Cholesteryl oleyl ether was used as a marker of the liposomal lipid phase. In addition, [$^{14}$C]sucrose was incorporated in the internal aqueous phase to study the integrity of the liposomes. Double-radiolabelled immunoliposomes and non-targeted liposomes were administered i.p. to OVCAR-3-bearing nude mice at different times (day 7, 14 or 21) after tumour cell inoculation. On all days of examination, the cell binding of immunoliposomes was much higher compared with non-targeted liposomes (see Table I). About 5% of the injected $^3$H-liposomal dose was found in spleen, liver and blood, 5 h after injection. The integrity of the liposomes with respect to release of the aqueous contents was also investigated. Release of sucrose from the liposomes will be detected as an increase in the $^3$H/$^{14}$C ratio of the injected liposome preparation. It was calculated that the loss of sucrose label from both immunoliposomes and non-targeted liposomes present in the peritoneal cavity was minimal (<15%) 5 h after injection, indicating that the bilayer structure of the liposomes remained intact (results not shown).

**Figure 1** *In vitro* cell binding of DXR-immunoliposomes. OVCAR-3 cells were incubated with DXR-immunoliposomes (○ and ●) or unconjugated DXR-liposomes (□ and ■) for 30 (○, □) or 90 min (●, ■) at 37°C. After washing the cells to remove unbound liposomes, cell-associated radioactivity was determined as described under Materials and methods. The degree of cell binding is expressed as the estimated amount of liposomes (nmol TL) bound per $10^6$ cells. The Fab′/lipid ratio of the immunoliposomes was 12 µg OV-TL3 per µmol TL. The outcome of a representative experiment is shown.

**Figure 2** Growth of OVCAR-3 in NMRI nude mice. Mice were injected i.p. with $2\times10^7$ OVCAR-3 cells (day 0). At days 7, 14 and 21 after tumour cell inoculation, mice were sacrificed for determination of tumour load [ascitic tumour cells (▩) and solid tumour (□)]. Values represent the mean tumour load±s.d. of 8–12 animals.

**Figure 3** Effect of DXR-immunoliposomes on growth of OVCAR-3 ovarian carcinoma *in vivo*. OVCAR-3 tumour-bearing athymic nude mice were treated with a single i.p. injection on day 7 after tumour cell inoculation. DXR-immunoliposomes ( ▭ ) or unconjugated DXR-liposomes (▩) were administered in a volume of about 0.5 ml. Control mice received PBS. The i.p. tumour load was determined on days 21 and 22 after tumour cell inoculation. Values represent the mean tumour load of 3–4 animals; for the untreated group six animals were used. The Fab′/lipid ratio of immunoliposomes was 20 μg OV-TL3 per μmol TL. When the tumour load was less than 0.5 g, the determined weight was mainly caused by cells other than tumour cells (e.g. lymphocytes).

To investigate the question, whether the fate of the drug, doxorubicin, parallels the fate of the liposomes, the (immuno)liposomes were labelled with [$^{14}$C]DXR and administered i.p. on day 7 after inoculation of the OVCAR-3 cells. Radioactivity associated with the tumour cells was determined 5 h and 24 h after injection. As shown in Table II, the percentage cell-bound [$^{14}$C]DXR was not significantly different after administration of non-targeted [$^{14}$C]DXR-liposomes or [$^{14}$C]DXR-immunoliposomes. Apparently, non-targeted liposomes are as effective as the immunoliposomes in the delivery of DXR to the cancer cells. The extent of DXR associated with the tumour cells after administration of non-targeted [$^{14}$C]DXR-liposomes was much higher than expected on the basis of the levels of cell-associated liposomal $^{3}$H label (see Tables I and II). This observation suggested to us that part of the tumour cell-associated $^{14}$C-DXR must be attributed to binding of [$^{14}$C]DXR released from the liposomes. To study this possibility, the binding of free DXR to the peritoneal tumour cells was also examined. Free [$^{14}$C]DXR was administered i.p. in ascitic OVCAR-3 tumour-bearing mice on day 21 after tumour cell inoculation. Figure 4 shows that [$^{14}$C]DXR is disappearing from the peritoneal cavity at a slow rate. Even 24 h after injection of [$^{14}$C]DXR 29 ± 4% of the injected dose was recovered from the peritoneal cavity. In line with the earlier suggestion, most of the injected amount of DXR appeared to be associated with the OVCAR-3 cells.

**Table II** Doxorubicin levels associated with peritoneal OVCAR-3 cells after i.p. administration of [$^{14}$C]DXR-immunoliposomes

| | *Injected $^{14}$C-DXR dose (%) associated wih the tumour cells* | |
|---|---|---|
| *Time after administration (h)* | *Unconjugated liposomes* | *Immuno-liposomes* |
| 5 | 52 ± 2 | 58 ± 7 |
| 24 | 34 ± 8 | 35 ± 2 |

[$^{14}$C]-DXR-(immuno)liposomes were administered i.p. into athymic nude mice on day 7 after tumour cell inoculation. Radioactivity was determined 5 h and 24 h after administration as described under Materials and methods. The Fab′/lipid ratio of the immunoliposomes was 19 μg OV-TL3 per μmol Tl. The mean tumour load was 1.3 ± 0.3 g. Data represent the mean ± s.d. of five animals.

**Figure 4** Recovery of free $^{14}$C-DXR in the peritoneal cavity of OVCAR-3-bearing nude mice. $^{14}$C-DXR (40 μg per mouse, i.e. approximately 1.5 mg DXR kg$^{-1}$) was administered i.p. on day 21 after tumour cell inoculation in a volume of 0.5 ml. Results are expressed as the % of the injected dose $^{14}$C-DXR present in the peritoneal cavity (○) and associated with tumour cells (●). Values represent the mean tumour load of 4–5 animals.

### Discussion

The study presented in this paper is concerned with the use of immunoliposomes (antibody-targeted liposomes) as carriers for doxorubicin (DXR) for i.p. chemotherapy of ovarian carcinoma. As a prelude to assessing the anti-tumour activity of DXR-containing OV-TL3 immunoliposomes towards *in vivo* growing human ovarian cancer cells (OVCAR-3), we first started to evaluate the cytotoxic effects and tumour-cell binding of DXR-immunoliposomes towards *in vitro* cultured OVCAR-3 cells. The *in vitro* cell growth inhibition of DXR encapsulated in immunoliposomes was far (200-fold) superior to DXR encapsulated in similar non-targeted liposomes (lacking the specific antibody fragments), and almost equalled the efficacy of the free drug. In line with these results, effective tumour cell binding was obtained with OV-TL3 DXR-immunoliposomes and not with non-targeted DXR-liposomes. The cell binding data are in the same

**Table I** Effect of i.p. tumour load on the degree of tumour cell binding of immunoliposomes

| *Tumour load (g)* | *Injected $^{3}$H dose (%) associated with the tumour cells* | |
|---|---|---|
| *Day after tumour cell inoculation* | *Unconjugated liposomes* | *Immunoliposomes* |
| 1.0 ± 0.1 (day 7) | 17 ± 10 | 42 ± 13* |
| 2.7 ± 0.4 (day 14) | 6 ± 3 | 74 ± 6** |
| 4.6 ± 0.4 (day 21) | 6 ± 1 | 88 ± 13** |

(Immuno)liposomes containing [$^{3}$H]-cholesteryl oleyl ether were injected i.p. into athymic nude mice, 7, 14 or 21 days after tumour cell inoculation. Radioactivity was determined 5 h after administration as described under Materials and methods. The Fab′/lipid ratio of the immunoliposomes was 30 μg OV-TL3 per μmol TL. Data represent the mean ± s.d. of 4–6 animals. *$P < 0.05$; **$P < 0.0001$ (immunoliposomes *vs* unconjugated liposomes on the same day after tumour cell inoculation).

range as those reported earlier obtained in cell binding experiments with OV-TL3 immunoliposomes without DXR (Nässander *et al.*, 1995), indicating that inclusion of DXR does not interfere with the cell binding of immunoliposomes.

As the combined *in vitro* results on target cell binding and cytotoxicity of DXR-(immuno)liposomes strongly suggest that specific binding to the cells is conferring greater tumour cytotoxicity, studies were initiated to evaluate the i.p. anti-tumour effects *in vivo*. The i.p. growing OVCAR-3 tumour was used because of resemblance to human *in situ* ovarian carcinoma in terms of morphology, histology and synthesis of tumour-specific antigens (Hamilton *et al.*, 1984). The characteristics of ovarian carcinoma, i.e. it usually remains within the peritoneal cavity and rarely disseminates to distant body sites, were confirmed. In an earlier study we reported that OV-TL3 immunoliposomes bind rapidly and efficiently (over 80% of the injected dose on day 21 after tumour cell inoculation) in this xenograft model. *In vivo* target cell binding experiments confirmed these data and showed that the observed degree of tumour cell binding is dependent on the day of administration (i.e. the tumour load). As shown in Figure 3, both DXR-immunoliposomes and non-targeted DXR-liposomes induced a pronounced anti-tumour effect. However, no difference in anti-tumour activity between the targeted and non-targeted formulation was observed. Thus, despite the observation of specific binding of the immunoliposomes to the tumour cells, differences in anti-tumour response exerted by the DXR-immunoliposomes and non-targeted DXR-liposomes were observed only *in vitro* and not *in vivo*. One likely explanation for this discrepancy is the occurrence of considerable drug leakage from the administered specific and non-specific DXR-liposomes induced by the peritoneal environment. Rapid, premature drug loss may have overshadowed the occurrence of specific immunoliposome-mediated anti-tumour effects. Studies with radiolabelled DXR showed that practically all DXR recovered from the peritoneal cavity was associated with the tumour cells after i.p. administration of free DXR (Figure 4). Likewise, DXR leaked from DXR-immunoliposomes or non-targeted DXR-liposomes present in the peritoneal cavity will rapidly associate with the tumour cells. The observation that the non-targeted liposomes, which show little cell binding, are able to deliver as much DXR to the target cells as the specific immunoliposomes (Table II) strongly indicates that a large part of the liposomal DXR content is rapidly lost from the non-targeted liposomes. Considering the identical lipid composition, it is reasonable to assume that the extent of DXR leakage was similar for the targeted liposomes. Therefore, leakage and subsequent rapid association with the tumour cells is likely to explain the absence of any differences in anti-tumour response exerted by the immunospecific and non-specific DXR-liposomes and makes it difficult to discriminate between DXR anti-tumour activity induced by cell-bound and non cell-bound liposomes. The fact that differences in tumour cell growth inhibition were observed *in vitro* and not *in vivo* probably relates to the different composition of the incubation medium compared with ascites and the much shorter incubation time (30 min) compared with the long residence time in the peritoneal cavity of the mice.

Considering that the majority of the injected DXR molecules associate rapidly with the OVCAR-3 cells after i.p. injection, it is clear that the anti-tumour activity of free DXR is not easily improved by (immuno)liposome-mediated delivery. Other cytostatics with less affinity for the tumour cells should be considered as more promising candidates for immunoliposome-mediated delivery in this tumour model. After cell binding of immunoliposomes, the encapsulated drug can enter the cell via different routes. Firstly, the immunoliposomes can be taken up by endocytosis. However, Nässander *et al.* (1995) showed that internalisation by these tumour cells takes place only to a low degree. Secondly, the liposomal contents can be released slowly after tumour cell binding, providing high DXR concentrations near the tumour cell surface. The results presented in this paper indicate that premature drug leakage occurring before binding of the immunoliposomes to the tumour cells plays a more important role in the mechanism of DXR uptake in this tumour model than leakage from cell-bound immunoliposomes. One way to improve the situation for DXR-immunoliposomes in this model is to prevent premature drug leakage by manipulation of the lipid bilayer composition. By the selection of phospholipids with higher phase transition temperatures, more stable DXR-liposomes can be prepared. However, the more rigid DXR-immunoliposomes were not nearly as effective as free DXR and the more fluid-type immunoliposomes used in this study (results not shown). This would indicate to us that cell-bound rigid immunoliposomes do not sufficiently release the encapsulated drug. To improve the therapeutic availability of the entrapped drug, triggered destabilisation of cell-bound rigid immunoliposomes seems a better option. After binding of stable DXR-immunoliposomes to the tumour cells and clearance of unbound DXR-liposomes from the peritoneal cavity, destabilisation by, for example, raising the temperature (then temperature-sensitive liposomes should be used, e.g. Yatvin *et al.*, 1978; Maruyama *et al.*, 1993), or lowering the pH (pH-sensitive liposomes, reviewed by Torchilin *et al.*, 1993) might lead to efficient tumour cell kill.

In summary, the *in vitro* cell growth inhibition of DXR encapsulated in OV-TL3 immunoliposomes is superior to that of non-targeted DXR-liposomes (lacking the specific antibody). In tumour-bearing nude mice, however, no difference in anti-tumour effect could be determined between targeted and non-targeted DXR-liposomes. Our results indicate that premature DXR leakage from (immuno)liposomes and subsequent DXR association with the tumour cells, explains why no significant differences in anti-tumour activity between DXR-immunoliposomes and non-targeted DXR-liposomes were observed. Further experiments will be focused on the *in vitro* and *in vivo* evaluation of stable, temperature-sensitive liposomes, which rapidly release their contents upon raising the temperature to about 42°C.

### Abbreviations

CHOL, cholesterol; DMEM, Dulbecco's modified Eagle's medium; DTT, dithiothreitol; DXR, doxorubicin; EPC, egg-phosphatidylcholine; EPG, egg-phosphatidylglycerol; FCS, fetal calf serum; Hepes, 4-(2-hydroxyethyl)-1-piperazineethanesulphonic acid; MPB-PE, N-[4-(p-maleimidophenyl)butyryl] phosphatidylethanolamine; PBS, phosphate-buffered saline; PE, phosphatidylethanolamine; SRB, sulforhodamine-B; TCA, trichloroacetic acid; TL, total lipid (phospholipid + cholesterol).

### Acknowledgements

We wish to thank Dr UK Nässander, MM Slobbe, C Moolenbeek, PJ van Schaaik and Ms D Kegler for their contributions. The gifts of the monoclonal antibody OV-TL3 from Professor Dr SO Warnaar (Centocor Europe BV), doxorubicin from Pharmachemie and phospholipids from Natterman Phospholipid GmbH were greatly appreciated. This work was supported by the Dutch Cancer Society, project no. IKMN 90-17.

### References

BOERMAN O, MASSUGER L, MAKKINK K, THOMAS C, KENEMANS P AND POELS L. (1990). Comparative *in vitro* binding characteristics and biodistribution in tumor-bearing athymic mice of anti-ovarian carcinoma monoclonal antibodies. *Anticancer Res.*, **10**, 1289–1296.

DELGADO G, POTKUL RK, TREAT JA, LEWANDOWSKI GS, BARTER JF, FORST D AND RAHMAN A. (1989). A phase I/II study of intraperitoneally administered doxorubicin entrapped in cardiolipin liposomes in patients with ovarian cancer. *Am. J. Obstet. Gynecol.*, **160**, 812–819.

DEPPE G, MALVIYA V, DECKER D, EVANS L, YOUNG J AND SCHILCHER B. (1985). A preliminary report on the toxicity and pharmacokinetics of combination systemic and intraperitoneal chemotherapy in advanced epithelial ovarian cancer. *Gynecol. Oncol.*, **20,** 269.

FISKE CH AND SUBBAROW Y. (1925). The colorimetric determination of phosphorus. *J. Biol. Chem.*, **66,** 375–400.

FORSSEN EA AND TOKES ZA. (1981). Use of anionic liposomes for the reduction of chronic doxorubicin-induced cardiotoxicity. *Proc. Natl Acad. Sci. USA*, **78,** 1873–1877.

HAMILTON TC, YOUNG RC, MCKOY WM, GROTZINGER KR, GREEN JA, CHU EW, WHANG-PENN J, ROGAN AM, GREEN WR AND OZOLS RF. (1983). Characterization of a human ovarian carcinoma cell line (NIH:OVCAR-3) with androgen and estrogen receptors. *Cancer Res.*, **43,** 5379–5389.

HAMILTON TC, YOUNG RC, LOUIE KG, BEHRENS BC, MCKOY WM, GROTZINGER KR AND OZOLS RF. (1984). Characterization of a xenograft model of human ovarian carcinoma which produces ascites and intraabdominal carcinomatosis in mice. *Cancer Res.*, **44,** 5286–5290.

HARAN G, COHEN R, BAR LK AND BARENHOLZ Y. (1993). Transmembrane ammonium sulphate gradients in liposomes produce efficient and stable entrapment of amphiphatic weak bases. *Biochim. Biophys. Acta*, **1151,** 201–205.

MARKMAN M. (1991). Intraperitoneal chemotherapy. *Semin. Oncol.*, **18,** 248–254.

MARKMAN M, HAKES T, REICHMAN B, HOSKINS W, RUBIN S, JONES W, ALMADONES L AND LEWIS JL. (1989). Intraperitoneal therapy in the management of ovarian carcinoma. *Yale J. Biol. Med.*, **62,** 393–403.

MARTIN FJ AND PAPAHADJOPOULOS D. (1982). Irreversible coupling of immunoglobulin fragments to preformed vesicles. *J. Biol. Chem.*, **257,** 286–288.

MARUYAMA K, UNEZAKI S, TAKAHASHI N AND IWATSURU M. (1993). Enhanced delivery of doxorubicin to tumour by long-circulating thermosensitive liposomes and local hyperthermia. *Biochim. Biophys. Acta*, **1149,** 209–216.

MASSUGER LFAG, KENEMANS P, CLAESSENS RAMJ, VERHEIJEN RHM AND CORSTENS FHM. (1991). Detection and localization of ovarian cancer with radiolabelled monoclonal antibodies. *Eur. J. Obstet. Gynecol. Reprod. Biol.*, **41,** 47–63.

MOSELEY KR, BATTAILE A, KNAPP RC AND HAISMA HJ. (1988). Localization of radiolabelled $F(ab')_2$ fragments of monoclonal antibodies in nude mice bearing intraperitoneally growing human ovarian cancer xenografts. *Int. J. Cancer*, **42,** 368–372.

NÄSSANDER UK, STEERENBERG PA, POPPE H, STORM G, POELS LG, DE JONG WH AND CROMMELIN DJA. (1992). *In vivo* targeting of OV-TL3 immunoliposomes to ascitic ovarian carcinoma cells (OVCAR-3) in athymic nude mice. *Cancer Res.*, **52,** 646–653.

NÄSSANDER UK, STEERENBERG PA, DE JONG WH, VAN OVERVELD WOWM TE, BOEKHORST CME, POELS LG, JAP PHK AND STORM G. (1995). Design of immunoliposomes directed against human ovarian carcinoma. *Biochim. Biophys. Acta*, **1235,** 126–139.

OZOLS RF, YOUNG RC, SPEYER JL, SUGARBAKER PH, GREENE R, JENKINS J AND MYERS CE. (1982). Phase I and pharmacological studies of adriamycin administered intraperitoneally to patients with ovarian cancer. *Cancer Res.*, **42,** 4265–4269.

POELS LG, PETERS D, VAN MEGEN Y, VOOIJS GP, VERHEYEN RNM, WILLEMEN A, VAN NIEKERK CC, JAP PHK, MUNGYER G AND KENEMANS P. (1986). Monoclonal antibody against human ovarian tumor-associated antigens. *J. Natl Cancer Inst.*, **76,** 781–791.

POOL GL, FRENCH ME, EDWARDS RA, HUANG L AND LUMB RH. (1982). Use of radiolabeled hexadecyl cholesteryl ether as a liposome marker. *Lipids*, **17,** 445–452.

RUBINSTEIN LV, SHOEMAKER RH, PAULL KD, SIMON RM, SKEHAN P, SCUDIERO DA, MONKS A AND BOYD MR. (1990). Comparison of *in vitro* anticancer-drug-screening data generated with a tetrazolium assay versus a protein assay against a diverse panel of human tumor cell lines. *J. Natl Cancer Inst.*, **82,** 1113–1118.

RYGAARD J AND FRIIS CW. (1974). The husbandry of mice with congenital absence of the thymus (nude mice). *Z. Versuchstierk. Bd.*, **16,** 1–10.

SINGH M, GHOSE T, MEZEI M AND BELITSKY P. (1991). Inhibition of human renal cancer by monoclonal antibody targeted methotrexate-containing liposomes in an ascites tumour model. *Cancer Lett.*, **56,** 97–102.

SKEHAN P, STORENG R, SCUDIERO DA, MONKS A, MCMAHON J, VISTICA D, WARREN JT, BOKESH H, KENNEY S AND BOYD MR. (1990). New colorimetric cytotoxicity assay for anticancer-drug-screening. *J. Natl Cancer Inst.*, **82,** 1107–1112.

STEIN Y, HALPERIN G AND STEIN O. (1980). Biological stability of $[^3H]$cholesteryl oleyl ether in cultured fibroblasts and intact rat. *FEBS Lett.*, **111,** 104–106.

STORM G, VAN BLOOIS L, BROUWER M AND CROMMELIN DJA. (1985). The interaction of cytostatic drugs with adsorbents in aqueous media. *Biochim. Biophys. Acta*, **818,** 343–351.

STRAUBINGER RM, LOPEZ NG, DEBS RJ, HONG K AND PAPAHADJOPOULOS D. (1988). Liposome-based therapy of human ovarian cancer: parameters determining potency of negatively charged and antibody-targeted liposomes. *Cancer Res.*, **48,** 5237–5245.

TORCHILIN VP, ZHOU F AND HUANG L. (1993). pH-sensitive liposomes. *J. Lipid Res.*, **3,** 201–255.

WESSEL D AND FLÜGGE UI. (1984). A method for the quantitative recovery of protein in dilute solution in the presence of detergents and lipids. *Anal. Biochem.*, **138,** 141–143.

WISH L, FURTH J AND STOREY RH. (1950). Direct determination of plasma cell and organ-blood volumes in normal and hypervolemic mice. *Proc. Soc. Exp. Biol. N.Y.*, **74,** 644–648.

YATVIN MB, WEINSTEIN JN, DENNIS WH AND BLUMENTHAL R. (1978). Design of liposomes for enhanced local release of drugs by hyperthermia. *Science*, **202,** 1290–1293.